# 

**Published:** 1872-06

**Authors:** 


### CONTENTS:

ORIGINAL COMMUNICATIONS:
ARTICLE I. Memoranda of Cases of Cerebro-Spinal Meningitis......... 321
ART. II. Nux Vomica in Dyspepsia. 331
ART. III. Puerperal Convulsions.... 335

SELECTIONS:
On the Determining Cause of Labor. 339
On the Growth of the Nails as a Prognostic Indication in Cerebral Paralysis.......... 344
On the Diagnosis of Anæmic Murmurs 347
Amnesic and Ataxic Aphasia, etc.... 352
Mrs. Winslow's Soothing Syrup—a Poison .......... 358
Effect of Menstrual Disorders upon the Vascularity and Nutrition of the Intra-ocular Structures .......... 361
Clinic of Dr. Gregory at St. Louis Sisters' Hospital.......... 364
An Act to prevent the Sale of Drugs or Medicines designed to procure Criminal Abortion .......... 366

REPORTS OF SOCIETIES.
Indiana State Medical Society....... 367

EDITORS' BOOK TABLE.......... 378
EDITORIAL.......... 380
LOOT.......... 382

